# Congenital LMNA-Related Muscular Dystrophy in Paediatrics: Cardiac Management in Monozygotic Twins

**DOI:** 10.3390/ijms25115836

**Published:** 2024-05-27

**Authors:** Patricia Martínez Olorón, Iosune Alegría, Sergi Cesar, Bernat del Olmo, Estefanía Martínez-Barrios, Laura Carrera-García, Daniel Natera-de Benito, Andrés Nascimento, Oscar Campuzano, Georgia Sarquella-Brugada

**Affiliations:** 1 Unidad de Cardiología Infantil, Hospital Universtario de Navarra, 31008 Pamplona, Spain; josune.alegria.echauri@navarra.es; 2 Departamento de Pediatría, Facultad de Ciencias de la Salud, Universidad Pública de Navarra, 31006 Pamplona, Spain; 3 Pediatric Arrhythmias, Inherited Cardiac Diseases and Sudden Death Unit, Hospital Sant Joan de Déu, 08950 Esplugues de Llobregat, Spain; sergio.cesar@sjd.es (S.C.); estefania.martinez@sjd.es (E.M.-B.); georgia@brugada.org (G.S.-B.); 4 Arrítmies Pediàtriques, Cardiologia Genètica i Mort Sobtada, Malalties Cardiovasculars en el Desenvolupament, Institut de Recerca Sant Joan de Déu, 08950 Esplugues de Llobregat, Spain; 5 Institut d’Investigació Biomèdiques de Girona (IDIBGI-CERCA), 17190 Salt, Spain; bdelolmo@gencardio.com (B.d.O.); oscar@brugada.org (O.C.); 6 Centro Investigación Biomédica en Red-Cardiovascular (CIBERCV), Instituto de Salud Carlos III, 28029 Madrid, Spain; 7 Neuromuscular Unit, Department of Neurology, Hospital Sant Joan de Déu, 08950 Barcelona, Spain; laura.carrera@sjd.es (L.C.-G.); daniel.natera@sjd.es (D.N.-d.B.); andres.nascimento@sjd.es (A.N.); 8 Investigación Aplicada en Enfermedades Neuromusculares Neurociències, Institut de Recerca Sant Joan de Déu, 08950 Esplugues de Llobregat, Spain; 9 Instituto Nacional de Investigación Biomédica de Enfermedades Raras (CIBERER), Instituto de Salud Carlos III, 28029 Madrid, Spain; 10 Medical Science Department, School of Medicine, Universitat de Girona, 17003 Girona, Spain; 11 Pediatrics Department, School of Medicine, Universitat de Barcelona, 08007 Barcelona, Spain

**Keywords:** sudden cardiac death, laminopathies, arrhythmias, muscular dystrophy, genetic diagnostic

## Abstract

Pathogenic variants in *LMNA* have been associated with a wide spectrum of muscular conditions: the laminopathies. *LMNA*-related congenital muscular dystrophy is a laminopathy characterised by the early onset of symptoms and often leads to a fatal outcome at young ages. Children face a heightened risk of malignant arrhythmias. No established paediatric protocols for managing this condition are available. We review published cases and provide insights into disease progression in two twin sisters with *LMNA*-related muscular dystrophy. Our objective is to propose a cardiac surveillance and management plan tailored specifically for paediatric patients. We present a family of five members, including two twin sisters with *LMNA*-related muscular dystrophy. A comprehensive neuromuscular and cardiac work-up was performed in all family members. Genetic analysis using massive sequencing technology was performed in both twins. Clinical assessment showed that only the twins showed diagnoses of *LMNA*-related muscular dystrophy. Follow-up showed an early onset of symptoms and life-threatening arrhythmias, with differing disease progressions despite both twins passing away. Genetic analysis identified a de novo rare missense deleterious variant in the *LMNA* gene. Other additional rare variants were identified in genes associated with myasthenic syndrome. Early-onset neuromuscular symptoms could be related to a prognosis of worse life-threatening arrhythmias in *LMNA* related muscular dystrophy. Being a carrier of other rare variants may be a modifying factor in the progression of the phenotype, although further studies are needed. There is a pressing need for specific cardiac recommendations tailored to the paediatric population to mitigate the risk of malignant arrhythmias.

## 1. Introduction

The *LMNA* gene (Gene ID:4000; HGNC: 6636) is located at chromosome 1 (1q22) and encodes the lamin A/C protein. This protein is a key component of the nuclear lamina, a matrix of proteins situated next to the inner nuclear membrane. Deleterious variants in the *LMNA* gene give rise to a spectrum of muscular dystrophies characterised by variable dilated cardiomyopathy, collectively referred to as laminopathies. Among these conditions are Emery–Dreifuss muscular dystrophy, limb-girdle muscular dystrophy 1B, and *LMNA*-related congenital muscular dystrophy (L-CMD). These laminopathies often exhibit overlapping clinical features, prominently featuring muscle weakness and both cardiac structural alterations and conduction anomalies [1].

L-CMD is one of the most severe subtypes, accounting for approximately 6% of all congenital muscular dystrophies. It was first reported over 15 years ago, due to de novo deleterious variants in the *LMNA* gene [2]. L-CMD typically has an early onset, appearing during infancy. Affected individuals have muscular weakness that often hinder the achievement of motor milestones such as the ability to hold up heads, sit, or walk. Death tends to occur at a young age due to a heightened risk of respiratory insufficiency and cardiac arrhythmogenic episodes [3].

To date, only two large series in paediatric populations have been published, showing an autosomal dominant pattern of inheritance and highlighting malignant arrhythmias as the main cause of decease [4,5]. Our group proposed the first cardiac algorithm for the early identification and prevention of malignant arrhythmias in paediatric cases [4]. In addition, we reported a comprehensive genetic analysis in order to unravel novel rare variants in other genes related to neuromuscular diseases explaining potential differences in the time of onset and/or time-evolution in L-CMD cases [6]. The revaluation of rare variants, especially if previously classified with an ambiguous role, should be performed periodically until a definite role in muscular dystrophies has been clarified [7]. Here, we report a complete clinical diagnosis and late follow-up as well as a comprehensive update genetic analysis in two monozygotic twins diagnosed with L-CMD.

## 2. Results

**Figure 1:**
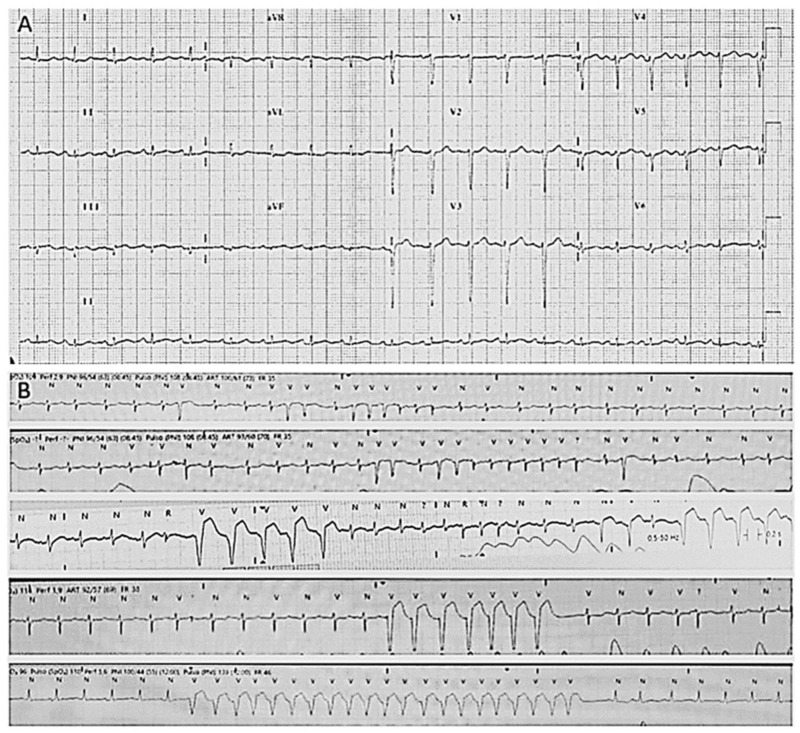
**Electrocardiograms of index case.** (**A**) Basal electrocardiogram with diminution of voltages. (**B**) Atrial fibrillation and non-sustained ventricular tachycardia.

**Figure 2:**
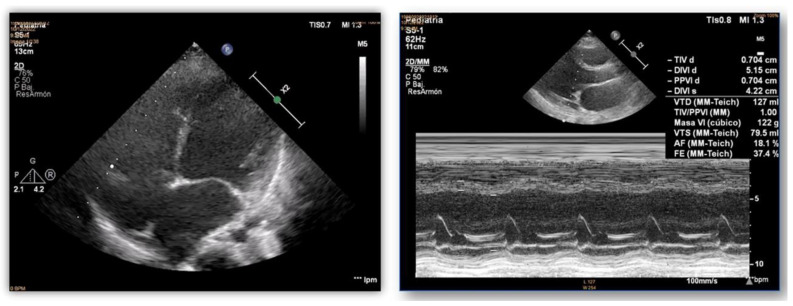
Echocardiograms of index case. Dilatation and poor systolic function of the left ventricle.

**Figure 3:**
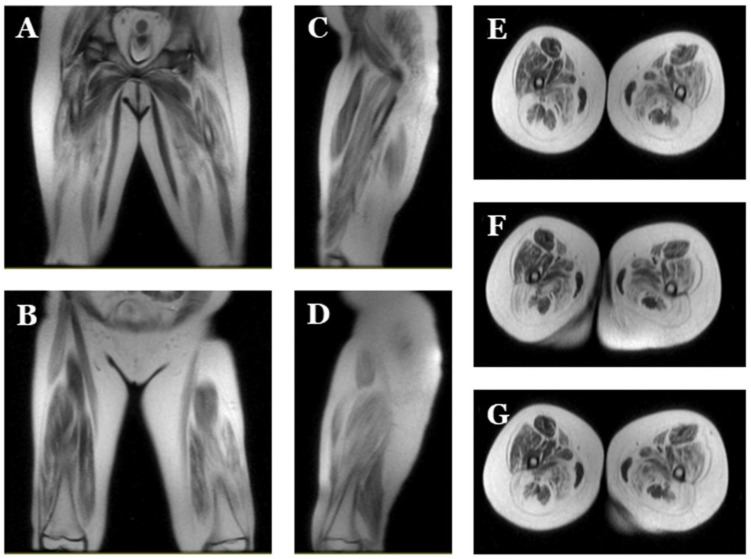
**Magnetic resonance images of index case.** Images of lower extremities showing diffuse bilateral atrophy with adipose infiltration. Coronal (**A**,**B**), sagittal (**C**,**D**) and axial (**E**–**G**) view.

**Figure 4:**
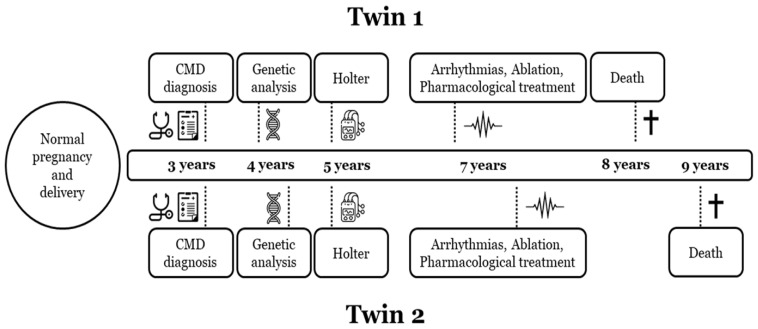
**Timeline disease in both twins.** Diagnosis and follow-up of both twin sisters (twin 1, index case). CMD, congenital muscular dystrophy. Symbol † means dead of the patient.

**Figure 5:**
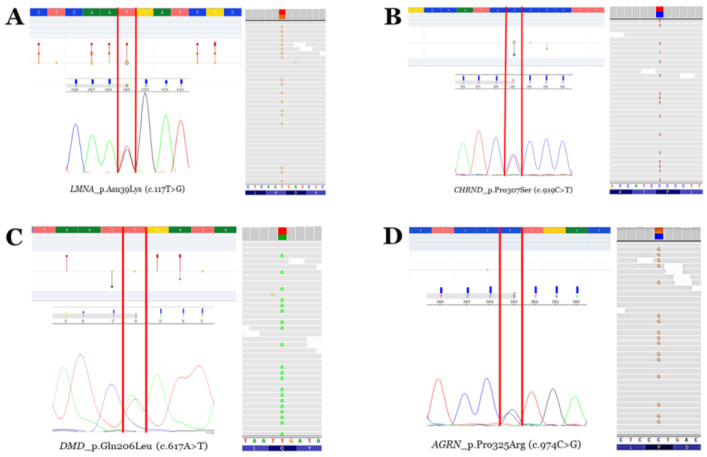
**Rare variants.** Electropherograms (Sanger—left bottom—and next-generation sequencing—right—modified from IGV software, version 2.11.7) and neighbouring zones—left top—of the four rare variants identified (modified from the Varsome database). Vertical red lines delimit the change in nucleotides leading to missense aminoacid variations. Red points mean pathogenic/likely pathogenic variants. Orange points mean variants of unknown significance. Green points mean likely benign/benign variants. (**A**) Rare variant p.N39K in the *LMNA* gene. (**B**) Rare variant p.P307S in the *CHRND* gene. (**C**) Rare variant p.Q206L in the *DMD* gene (antisense sequence in Sanger electropherogram). (**D**) Rare variant p.P325R in the *AGRN* gene.

**Figure 6:**
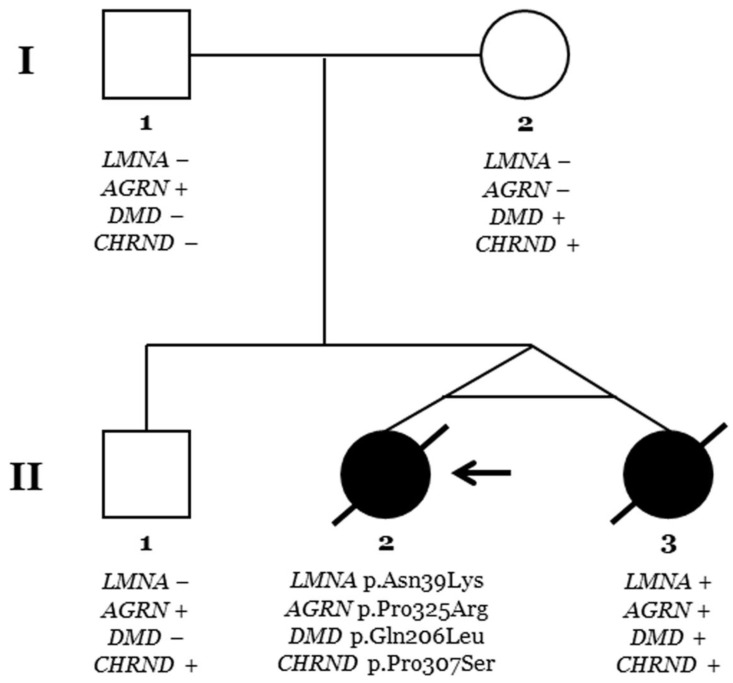
**Pedigree.** Generations are indicated on the left side. Each individual of direct family linage is identified with a number. Clinically affected patients are shown in black, and clinically unaffected patients are shown in white. Slash indicates deceased. The index case (II.2) is indicated with an arrow. The plus sign indicates a carrier of the genetic variant. The minus sign indicates a non-carrier of the genetic variant.

Limited numbers of diagnosed cases of L-CMD have been reported so far. Our group published a series of paediatric cases including monozygotic twins [4,6]. Here, we present a complete clinical assessment (Table 1; Figure 1, Figure 2 and Figure 3) and genetic analysis (Table 2) in twins and their parents and siblings, including a close follow-up (Figure 4). The genetic analysis identified four rare variants (Figure 5 and Figure 6). All these rare variants have been reclassified and updated with novel available data.

### 2.1. Clinical Data

Two monozygous twin sisters were delivered in week 34, with no alterations observed during pregnancy. The routine examination at birth was normal. Follow-up showed a progressive delay in the acquisition of motor milestones in both twins: sitting after 9 months and walking after 2 years (Table 1; Figure 1, Figure 2 and Figure 3). No diagnosis of any muscular alteration was performed then. However, on examination at 3 years old, both twins presented an increase in the base of support, hyperlordosis, flat feet, waddling gait, positive Gowers, generalised hypotonia and need for a wheelchair for long distances. After comprehensive assessment and additional tests requested, CMD was diagnosed in both twin sisters. At the moment of diagnosis, all the clinical findings were more severe in the first twin (II.2, index case) (Figure 6).

Concerning cardiac alterations, a normal ECG and echocardiography in both twins was observed at 3 years old. Two years after, a generalised decrease in electrical voltages was observed and a subcutaneous Holter monitor was implanted in both twins. The first twin (II.2, index case) presented an episode of paroxysmal supraventricular tachycardia at 7 years old. In the electrophysiological study (EPS), a polymorphic atrial tachycardia was discovered, and foci were ablated. One year later, continuous partially aberrated atrial fibrillation (AF) began, with hemodynamic repercussions (LVEF of 40%), with associated ventricular extrasystoles and bouts of non-sustained ventricular tachycardia. Pharmacological treatment did not control the arrhythmias, and EPS was performed with the ablation of atrial foci. A few hours after the EPS, another incessant atrial tachycardia began, with progressive deterioration and multi-organ failure. Unfortunately, she died at 8 years old (Figure 4). The second twin (II.3), exhibiting a mild neurological phenotype, showed normal cardiac function (LVEF of 60%) when her sister died. A few months later, she reported two self-limited episodes of dizziness, which the subcutaneous Holter identified as partially aberrated paroxysmal AF, and antiarrhythmic treatment was started. A week later, she presented an episode of ocular supraversion, deviation of the corner of the mouth, without loss of consciousness, leaving her with dysarthria and right hemibody focality. The CT angiography demonstrated obstruction of the left middle cerebral artery; endovascular treatment was performed for permeability and anticoagulant treatment was administrated. A cerebral infarction with femoral thrombosis, pallor, and coldness of the right leg was observed; revascularization was then achieved. Concomitantly, a mild but progressive impairment of contractile function developed, despite pharmacological treatment, with an LVEF of 40%. A complex ventricular extrasystole appeared 10 days after the cerebral infarction, and finally ventricular arrhythmia appeared that resulted in death at the age of 9 years (Figure 4).

### 2.2. Genetic Data

Genetic analysis using an NGS approach in the index case (II.2) identified four rare variants as potentially implicated in the phenotype (*LMNA*_p.Asn39Lys, *AGRN*_p.Pro325Arg, *DMD*_p.Gln206Leu, and *CHRND*_p.Pro307Ser), despite the rare variant in *LMNA* being suspected as the main cause of phenotype mainly due to previous variants in this gene associate with the same phenotype (Table 2; Figure 5). Genetic analysis in the other twin (II.3) identified the same four rare variants. Familial segregation showed all rare variants in heterozygosis. The rare variant in *LMNA* was de novo in both twins. Clinical assessment of the parents (I.1 and I.2) and brother (II.1) at the time of genetic analysis did not identify any clinical alteration concerning muscular dystrophy or similar phenotypes (Figure 6). Update of all four variants following ACMG recommendations identified novel data in the frequence population, reclassifying *CHRND*_p.Pro307Ser with an LB role due to an increase in MAF. The variants *AGRN*_p.Pro325Arg and *DMD*_p.Gln206Leu maintained an ambiguous role, and only *LMNA*_p.Asn39Lys were considered LP/P according to the available data (March 2024).

## 3. Discussion

In our study, we focused on the diagnosis and close follow-up of two monozygotic twin sisters diagnosed with L-CMD, an extremely rare case. The main cause of death reported so far in patients diagnosed with L-CMD are malignant arrhythmias and SCD [4,5]. However, only one previous study by our group has been published so far focused on cardiac management to reduce risk of cardiac events in a cohort of patients diagnosed with L-CMD [4].

Abnormal neuromuscular presentation was suspected in twins from their first periods of life, but the age of definite diagnosis was delayed until the age of motor development, as also reported in several cases [5,8]. The adoption of personalised measures in both twins prevented malignant arrhythmias in the early stages of the disease, even though, unfortunately, both twins ultimately passed away. The clinical evolution was similar but delayed in one of the twins, suggesting that the cause of disease was the deleterious variant in the *LMNA* gene, identified using an NGS approach, in concordance with recent studies focused on the diagnosis of neuromuscular diseases [9,10]. However, other genetic alterations may modify the temporal onset/progression of phenotypic expression. Our genetic analysis did not identify any genetic difference, as expected, due to monozygotic twins being genetically equal. However, other alterations in the genome and environmental interactions may be causes of temporal delays in phenotypic expression despite the similar outcomes in both twins.

The early onset of symptoms, as well as the aggressive phenotype identified in the twins, were in concordance with a previous report on L-CMD [8,11]. In these studies, no comprehensive genetic analysis was performed in order to identify any other rare variant responsible for the characteristic aggressive and rapid phenotype in L-CMD, as we have performed. The three rare variants identified in other genes currently associated with neuromuscular entities at the first time of analysis were classified as ambiguous, with a definite role despite suspected pathogenicity. The updated data showed that only two of these rare variants maintained an ambiguous role, and the third rare variant could be classified as benign. Therefore, we suspect the pathogenic variant in the *LMNA* gene to be the cause of the disease, but our hypothesis highlights that the combination of rare variants in different genes may lead to a more severe phenotype, or at least to the early onset of symptoms reported in L-CMD, as previously reported by our group [6]. This is in accordance with recent studies suggested that digenic models of inheritance may explain the incomplete penetrance/variable expressivity observed in neuromuscular entities [12].

### Limitations

L-CMD is a very rare and often underdiagnosed disease. The few reported cases worldwide of L-CMD impede the design and validation of cardiac management regimes in paediatric populations. The same limitation pertains to the confirmation of a conclusive genotype–phenotype correlation in rare variants in additional genes to *LMNA*. The additional variants identified seemed not to play a definite role in our studied family, but both in vivo and in vitro studies should be performed to clarify the pathophysiological mechanism associated with the progressive disabling phenotype associated with L-CMD in twins. In addition, we cannot discard the existence of additional genetic alterations located in other genes not included in the NGS custom panel used in our studied family. Due to the patients being monozygotic twins, performing whole-exome sequencing and/or whole-genome sequencing did not seem to be a genetically appropriate solution despite the comprehensive analysis of RNA sequencing and proteomic identification of variants associated with different disease evolutions. Finally, the contemporary classification of rare variants is based on available data, but periodic updates should be performed due to possible new data becoming available in the future that may modify current classifications.

## 4. Materials and Methods

This study was approved by the Ethics Committee of the Hospital Josep Trueta (Girona, Spain) and Hospital Sant Joan de Déu (Barcelona, Spain), following the Helsinki II declaration. Written informed consent to participate in this study was provided by the participants’ legal guardians. Written informed consent was also obtained from all relatives included in the study in order to facilitate both the clinical assessment and genetic analysis.

Clinical evaluation of all family members included a complete physical examination by a paediatric neurologist and paediatric cardiologist in children, as well as by a neurologist and cardiologist in the parents, as previously published [4,6]. Baseline cardiac work-up consisted of a transthoracic Doppler echocardiography, a 12-lead electrocardiogram (ECG), electrophysiological study (EPS), and the implantation of a long-term cardiac implantable loop recorder with a home monitoring system. The complete pedigree was obtained, including a history of neuromuscular and cardiac diseases, syncope, and unexplained deaths. All individuals were clinically assessed at Hospital Sant Joan de Déu (Barcelona, Catalonia, Spain).

Peripheral blood samples were obtained from each family member. Genomic DNA was analysed using next-generation sequencing (NGS). A total of 105 genes involved in neuromuscular diseases and the risk of malignant cardiac arrhythmias were analysed, as previously published [4,6]. All rare variants identified were classified following the current recommendations of the American College of Medical Genetics and Genomics (ACMG) [13], updated at the time of this report (March, 2024). Data were collected from the ClinVar (www.ncbi.nlm.nih.gov/clinvar/intro/, accessed on 2 May 2024), MasterMind (https://mastermind.genomenon.com, accessed on 2 May 2024), LitVar2 (www.ncbi.nlm.nih.gov/research/litvar2/, accessed on 2 May 2024), and Genome Aggregation Database-GnomAD (http://gnomad.broadinstitute.org/, accessed on 2 May 2024) databases. To avoid bias, three specialists independently reclassified the previously reported rare variants; all investigators discussed the data included in each item of the ACMG and came to a consensus regarding the final classification of all rare variants.

## 5. Conclusions

*LMNA*-related muscular dystrophy is caused by pathogenic variants in the *LMNA* gene leading to early onset and rapid development. Malignant arrhythmias are common in the early stages of life, increasing the risk of sudden death. We recommend personalised clinical management in paediatric populations, including remote home monitoring, preventive invasive management, and closer echocardiographic follow-up from early stages of life for the early diagnosis of life-threatening arrhythmias. Despite early management and close follow-up, aggressive evolution can be prolonged, but lethal outcome cannot be avoided with the currently available therapies. Additional genetic factors may play a key role in time-lap phenotype progression, representing a potential target for therapeutic approaches; however, translation into clinical paediatric practice should be performed with caution due to the lack of conclusive data.

## Figures and Tables

**Table 1:** **Clinical data.** DCM, dilated cardiomyopathy. F, Female. ICD, implantable cardioverter defibrillator. L-CMD, *LMNA*-related congenital muscular dystrophy. M, Male. N, No. ND, Nonclinical diagnosis. PM, pacemaker. Y, Yes.

Case	Gender	Neuromuscular Phenotype	Age Diagnosis	Early Onset	DCM	ICD /PM	Sudden Death	Variants
I.1	M	N	ND	N	N	N	N	*AGRN* p.Pro325Arg
I.2	F	N	ND	N	N	N	N	*DMD* p.Gln206Leu *CHRND* p.Pro307Ser
II.1	M	N	ND	N	N	N	N	*AGRN* p.Pro325Arg *CHRND* p.Pro307Ser
II.2	F	L-CMD	3 years	Y	N	N	Y	*LMNA* p.Asn39Lys *AGRN* p.Pro325Arg *DMD* p.Gln206Leu *CHRND* p.Pro307Ser
II.3	F	L-CMD	3 years	Y	N	N	Y	*LMNA* p.Asn39Lys *AGRN* p.Pro325Arg *DMD* p.Gln206Leu *CHRND* p.Pro307Ser

**Table 2:** **Genetic variants identified in both twins.** LB, likely benign. LP, likely pathogenic. NA, not available. VUS, variant of unknown significance.

Gene	Chromosome	Nucleotide Change	Protein Change	dbSNP	gnomAD	ClinVar	ACMG
*LMNA*	1q22	c.117T>G	p.Asn39Lys	NA	NA	VUS	LP
*AGRN*	1p36.33	c.974C>G	p.Pro325Arg	NA	NA	NA	VUS
*DMD*	Xp21.1	c.617A>T	p.Gln206Leu	rs994260472	NA	NA	VUS
*CHRND*	2q37.1	c.919C>T	p.Pro307Ser	rs142063328	0.15%	VUS	LB

## Data Availability

The datasets generated during and/or analysed during the current study are available from the corresponding author on reasonable request.
